# Soil characteristics and bacterial community characteristics of shelterbelts of different tree species in black soil region of China

**DOI:** 10.1038/s41598-025-92051-3

**Published:** 2025-03-13

**Authors:** Jun Zhang, Wei Jing, Ke Ji, Yong Zhang

**Affiliations:** 1 Shandong Key Laboratory of Eco-Environmental Science for Yellow River Delta, Shandong University of Aeronautics, 391 Huanghe Fifth Road, Binzhou, 256603 Shandong China; 2 China Railway No.4 Engineering Group Co., Ltd, Hefei, 230000 Anhui China

**Keywords:** Farmland shelterbelts, Afforestation, Agroforestry system, High throughput sequencing, Soil bacterial diversity, Forestry, Soil microbiology

## Abstract

To understand how surface soil characteristics and bacterial communities are affected by the establishment of farmland shelterbelts. Five types of shelterbelts in the mid-west of Heilongjiang Province China were selected for the study. The physicochemical characteristics and bacterial diversity of *Populus×xiaohei* monoculture (X), *Larix gmelinii* monoculture (L), *Pinus sylvestris* monoculture (Z), *Pinus sylvestris* and *Larix gmelinii* mixed forest (ZL), and *Fraxinus mandshurica* and *Larix gmelinii* mixed forest (SL), as well as in fallow land (CK), were measured and analyzed, respectively. Soil physicochemical characteristics and bacterial diversity (via high-throughput sequencing) were analyzed across 0–20 cm depths. Results showed that shelterbelts significantly altered soil characteristics: X increased moisture, ammonium nitrogen, and microbial biomass nitrogen but reduced aeration. ZL exhibited the highest bacterial richness and enhanced water-holding capacity, aeration, and nutrient retention (total organic carbon, nitrogen, phosphorus). ZL outperformed monocultures in promoting soil health, with available potassium (0–10 cm) and pH (10–20 cm) identified as key drivers of bacterial community variation. Unique genera like *Krasilnikovia* and *Rubrobacter* dominated shelterbelt soils, reflecting species-specific effects. Shelterbelts induced surface accumulation of nitrate-nitrogen, potassium, and microbial biomass carbon. Overall, *Pinus sylvestris* and *Larix gmelinii* mixed forests optimized soil structure, microbial diversity, and nutrient cycling, underscoring their ecological benefits for sustainable agroforestry. This study highlights the critical role of mixed forest shelterbelts in enhancing soil health and microbial biodiversity, which are essential for sustainable land management practices in the black soil region of China.

## Introduction

In recent years, older farmland shelterbelts planted decades ago in China have begun to show stand degradation in many areas, resulting in decreased effectiveness^1^. With the ongoing phenomenon of shelterbelt premature aging, current research primarily addresses altered growth conditions, including drought-mortality relationships^2^, soil microbial diversity^3^, and soil biogeochemical dynamics encompassing carbon^4^, nitrogen^5^, physical properties^6^, and water balance^7^.

Forest-type comparisons (monocultures^8^, mixed species^9,10^) have established baseline soil characteristics, yet microbial indicators^11–13^ better capture soil quality dynamics. Critical gaps persist in identifying drivers of soil changes within farmland shelterbelts, particularly across soil depths^14^. Emerging evidence demonstrates that mixed tree species enhance soil quality through improved aggregate stability and nutrient cycling^15,16^, with species selection and vertical soil stratification jointly shaping soil-bacterial relationships^17,18^. In this study, soil characteristics were measured and analyzed for five types of farmland shelterbelts (varying in tree species composition) and one plot of unforested land (fallow since the establishment of the forest shelterbelts) in the mid-western black soil area of Heilongjiang Province, China. We focused on this critical Three-North Shelterbelt region where wind erosion threatens both ecological security and grain production. By quantifying soil characteristics and examining correlations among them, the following questions were addressed: (1) How do mixed species shelterbelts differentially influence soil physicochemical propertie0s and bacterial community structure? and (2) What are the key soil factors driving the vertical stratification of bacterial communities in shelterbelt ecosystems, and how do these factors interact with tree species composition?

### Study sites and forest characteristics

**Fig. 1:**
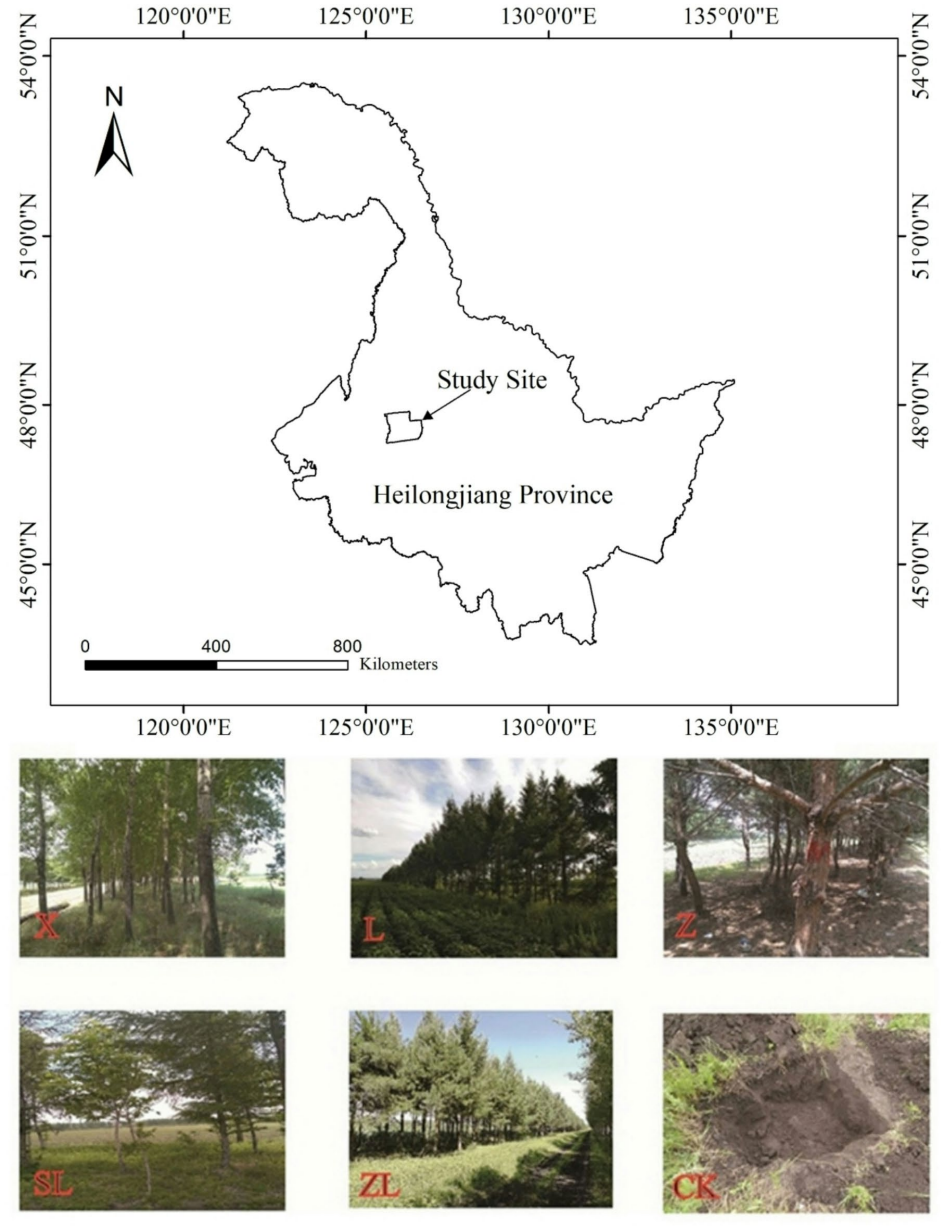
Location of the study area and photographs of the different study sites.

This study was carried out on the plains of Baiquan County, located in the midwest of Heilongjiang province, China (Fig. 1). The region has mostly black soils derived from yellow clay originating from quaternary lacustrine alluvial sediments, topsoil texture ranges from silty clay loam to clay loam^19^.

From July 9 to 24, 2018, three 20 m by 8 m repeated plots were set for each forest type in different sites, and three unplanted plots were added to make a total of 18 plots (125°18′638″E—125°51′18″E, 47°38′4″N—47°39′23″N). Among them, 5 forest types were *Populus×xiaohe*i pure forest (X), *Larix gmelinii* (Rupr.) Kuzen. pure forest (L), *Pinus sylvestris* var. *mongholica* Litv. pure forest (Z), *Pinus sylvestris* var. *mongholica* Litv. and *Larix gmelinii* (Rupr.) Kuzen. mixed forest (ZL), *Fraxinus mandshurica* Rupr. and *Larix gmelinii* (Rupr.) Kuzen. mixed forest (SL), and unforested field (CK). The soil types of these shelterbelts were primarily black soil, which is known for its high organic matter content and fertility.

The different types of study sites were located as close to as possible to one another, to minimize variation in soil characteristics across sites^20^. For each type of sample plot, basic forest characteristics were measured; these data are given in Table 1.

**Table 1:** Forest characteristics for each study site.

Site type	Plant age / a	Spacing / m	Height / m	DBH /cm	Altitude / m	Location
X	20	2 × 1.5	17.86 ± 3.66	16.64 ± 1.58	241 ~ 241	125°51’12.4"E-125°51’38.0"E, 47°38’22.8"N-47°38’26.2"N
L	20	2 × 1.5	9.44 ± 1.90	11.00 ± 1.15	239 ~ 267	125°48’59.7"E-125°49’03.5"E, 47°38’38.2"N-47°38’55.3"N
Z	20	2 × 1.5	8.18 ± 0.89	12.59 ± 1.30	248 ~ 251	125°51’37.9"E-125°51’41.4"E, 47°38’11.9"N-47°38’24.8"N
SL	20	2 × 3	9.76 ± 1.01	10.99 ± 1.16	242 ~ 252	125°49’03.3"E-125°49’06.4"E, 47°38’23.6"N-47°38’36.8"N
ZL	20	2 × 1.5	11.08 ± 1.11	13.96 ± 1.43	249 ~ 256	125°47’15.5"E-125°47’17.4"E, 47°39’09.6"N-47°39’30.5"N
CK	—	—	—	—	221 ~ 222	125°48’28.1"E-125°48’29.9"E, 47°38’35.6"N-47°38’36.4"N

X, L, Z, SL, and ZL refer to the different study sites planted with *Populus×xiaohei*, *Larix gmelinii* (Rupr.) Kuzen., *Pinus sylvestris* var. *mongholica* Litv., *Fraxinus mandshurica* Rupr. mixed with *Larix gmelinii* (Rupr.) Kuzen., *Pinus sylvestris* var. *mongholica* Litv. mixed with *Larix gmelinii* (Rupr.) Kuzen., respectively; CK refers to the unforested study site. The values in the table are the mean ± SD.

### Soil sample collection

From July 14 to 20, 2018, five sampling points were selected following an “S” shape in each sample plot; surface litter and any weeds were removed. Soil pit 1 m long, 1 m wide, and 20 cm deep were excavated at each sampling point. Each soil pit was divided into two layers, an upper layer (0–10 cm) and a lower layer (10–20 cm); samples were collected from the middle of each layer^3^. Two soil specimens per sampling point-layer combination were collected: (1) 100 g fresh soil for immediate moisture quantification and microbial DNA extraction, and (2) 500 g soil air-dried for subsequent physicochemical analyses. Thus, a total of 360 soil samples were collected (i.e., 18 plots × 5 points × 2 depths × 2 types = 360 initial samples ). Undisturbed soil samples were collected from each layer using a ring knife, weighed, and used to determine bulk density (BD), porosity, and other properties. Approximately 10 g of fresh soil was placed in an aluminum box, weighed immediately, and used to measure soil moisture (MCg). After removing impurities (e.g., roots, stones), the soil was sieved through a 2 mm mesh. Samples from five points within the same layer were mixed in equal volumes to create a 1 kg composite sample, which was labeled, air-dried, and transported to the laboratory for analysis. Similarly, a 200 g composite fresh soil sample was collected for each layer and plot, stored at 4℃ until testing. Finally, to ensure that the soils used for DNA extraction remained fresh, these soil samples were collected on the morning of July 21, 2018. From an undisturbed area near each sampling point, a sterile sampler was used to collect a 5 g sample from the middle of each soil layer; impurities, such as visible rocks and roots, were removed, and the sample was then passed through a sterile 2 mm sieve. Following the same protocol described above, a 5 g mixed fresh soil sample was obtained for each soil layer and sample plot; this was stored in a sterile centrifuge tube. Thus, a total of 36 mixed soil samples were collected, placed in a box with dry ice, and transported to the laboratory for DNA extraction^21^.

## Methods

### Measurement of soil physicochemical characteristics

The MCg was determined by drying the soil samples. Soil BD, saturation holding capacity (SHC), capillary holding capacity (CHC), and field holding capacity (FHC) were measured using the cutting ring method. The soil non-capillary porosity (NCP), capillary porosity (CP), total porosity (Pt), and aeration (Ae) were then calculated. A soil suspension was prepared (soil to water ratio of 1:2.5 w·v^− 1^), and the pH measured using a potentiometric pH meter^22^. The soil total organic carbon (TOC) and total nitrogen (TN) were determined using an elemental automatic CN-analyzer (Vario Max CN Macro Elemental Analyzer, Elementar Analysensysteme GmbH, Hanau, Germany) via the dry combustion method^23^. The ammonium nitrogen (AN) and nitrate-nitrogen (NN) were quantified using an AA3 automatic continuous segmented flow analyzer (AutoAnalyzer 3, SEAL Analytical GmbH, Norderstedt, Germany) after leaching with a 1 mol·L^− 1^ KCl solution^24^. The available phosphorus (AP) was determined by first extracting a sample in a 0.5 mol·L^− 1^ NaHCO_3_ solution and then analyzing the resultant color at 700 nm using a spectrophotometer. The total phosphorus (TP) was measured with the aid of a sulfuric acid-perchloric acid solution and Mo-Sb colorimetry. Following extraction with a 1 mol·L^− 1^ NH_4_OAC solution, the available potassium (AK) was measured using a flame photometer, while the total potassium (TK) was determined using a hydrofluoric acid-perchloric acid solution and flame photometry^25^. Finally, chloroform fumigation was used to quantify the microbial biomass carbon (MBC) and microbial biomass nitrogen (MBN)^26^. Soil samples were fumigated with ethanol-free chloroform for 24 h. After fumigation, the samples were extracted using 0.5 mol/L K_2_SO_4_. The conversion factors used to calculate MBC and MBN were 0.38 and 0.45, respectively.

### Soil bacterial DNA extraction, PCR amplification and sequencing

The total soil DNA was extracted from all 36 samples using a Soil DNA Kit (Omega M5635-02, Omega Bio-Tek Co., LTD, Norcross, GA, US) following the manufacturer’s instructions^27^. A Nanodrop-ND1000 ultraviolet spectrophotometer (Thermo Fisher Scientific, Wilmington, MA, USA) was used to assess the quantity and quality of the extracted DNA. The highly variable V3 ~ V4 region of the soil bacterial 16 S rDNA was amplified using a thermal circulation PCR system (Gene Amp 9700, ABI, Foster, CA, USA). The forward primer was 338 F (5′-barcode + ACTCCTACGGGAGGCAGCA-3′), and the reverse primer was 806R (5′-GGACTACHVGGGTWTCTAAT-3′)^28^. The PCR reaction mix and program were according to Zhang^3^. PCR amplicons were purified using an Axygen DNA gel kit (Axygen Biosciences, Union City, CA, USA) and quantified using the Quant-iT PicoGreen dsDNA Assay Kit (Invitrogen P7589, Carlsbad, CA, USA) and a microplate reader (BioTek, FLx800). Purified and quantified amplicon libraries were constructed according to sequencing requirements. The Illumina MiSeq platform with MiSeq Reagent Kit V3 (600 cycles) was used for paired-end 2 × 250 bp sequencing.

### Sequence analysis

The raw sequencing reads were processed using the QIIME (Quantitative Insights Into Microbial Ecology, V1.8.0, http://qiime.org) pipeline and R packages (Version 3.2.0)^28^. High-quality sequences were clustered into operational taxonomic units (OTUs) at 97% sequence identity using the UCLUST comparison tool^29^. The sequence with the highest abundance for each OTU was selected as representative of that OTU. The representative sequences were then BLASTed^30^ against the Silva database (Release 115, http://www.arb-silva.de) to obtain taxonomic information; a similarity of 0.999 was considered a match. To improve the analysis efficiency, and ensure its reliability and accuracy, OTUs with an abundance of less than 0.001% were excluded^31^, and the OTU abundance matrix then modified accordingly. Meanwhile, all samples were randomly resampled at the lowest observed sequencing depth (90%), to minimize differences caused by variation in sequencing depth across samples^32^.

### Statistical analysis

Analyses were performed using Microsoft Excel 2016 and SPSS 26.0, while Origin Pro2020Sr0 was used to create figures. To examine differences in soil physicochemical characteristics among treatments, one-way ANOVAs were implemented, followed by least significant difference (LSD) tests to compare means (α = 0.05)^33^. Venn diagrams were generated to visualize the number of OTUs shared among study sites, as well as unique to each site, using the R package “Venn Diagram”; diagrams were based on OTU occurrence and not relative abundance^34^. The Galaxy online analysis platform (http://huttenhower.sph.harvard.edu/galaxy/) was used to detect differentially abundant genera across study sites. A redundancy analysis (RDA) was performed using Canoco 5.0^35^ to examine relationships among soil characteristics, forest type, and the abundance of common soil bacterial genera.

## Results

### Soil physicochemical and microbial biomass characteristics of different types of sample plots

Soil physical characteristics were shown in Table 2. In the 0–10 cm soil layer (the “upper” soil layer), only the soil BD, MCg and Ae differed among study sites (*P* < 0.05; note significance assessed at *P* < 0.05 unless otherwise stated). However, in the 10–20 cm soil layer (the “lower” soil layer), most soil physical characteristics varied among sites except for soil BD, FHC, and CP.

**Table 2:** Soil physical characteristics in forest shelterbelts of different types.

Layer/cm	Sample plot	BD/g•cm^− 1^	SHC/%	CHC/%	FHC/%	NCP/%	CP/%	Pt/%	MCg/%		Ae/%
0–10	X	1.17 ± 0.03Cb	37.78 ± 4.17Aa	36.38 ± 3.75Aa	35.36 ± 3.07ABa	1.64 ± 0.61Aa	42.48 ± 4.66Aa	44.12 ± 5.14Ba	29.07 ± 0.76Aa		10.22 ± 4.89Ca
	L	1.33 ± 0.09ABa	35.13 ± 4.49Aa	33.10 ± 3.90Aa	32.17 ± 3.56Ba	2.62 ± 1.27Aa	43.80 ± 2.73Aa	46.43 ± 3.32ABa	20.52 ± 0.23Ea		18.95 ± 4.99ABa
	Z	1.26 ± 0.04ABCa	41.28 ± 1.87Aa	38.07 ± 1.21Aa	37.45 ± 1.30Aa	4.02 ± 1.12Aa	48.26 ± 1.08Aa	52.28 ± 1.00Aa	21.45 ± 1.09DEa		25.09 ± 1.81Aa
	SL	1.23 ± 0.04BCa	36.72 ± 4.19Aa	34.65 ± 3.10Aa	33.56 ± 2.45ABa	2.50 ± 1.43Aa	42.54 ± 3.12Aa	45.05 ± 4.34ABa	23.95 ± 1.18BCa		15.63 ± 3.81BCa
	ZL	1.29 ± 0.05ABa	38.42 ± 1.78Aa	35.86 ± 1.05Ab	34.40 ± 1.06ABb	3.26 ± 0.96Aa	46.47 ± 0.92Ab	49.74 ± 1.37ABa	22.41 ± 1.26CDa		20.64 ± 1.83ABb
	CK	1.35 ± 0.04Aa	35.39 ± 1.72Aa	33.57 ± 1.28Aa	32.76 ± 1.08ABa	2.42 ± 1.41Aa	45.47 ± 1.28Aa	47.90 ± 1.52ABa	24.32 ± 0.37Bb		14.90 ± 2.17BCa
	*P*	0.012	0.405	0.379	0.214	0.402	0.202	0.150	0.000		0.013
10–20	X	1.31 ± 0.06ABa	32.50 ± 2.38Ba	31.65 ± 2.14Ba	31.09 ± 2.04Ba	1.13 ± 0.53Ca	41.78 ± 3.75Ba	42.91 ± 4.09Ba	24.84 ± 2.78ABa		10.36 ± 5.10Ba
	L	1.37 ± 0.06Aa	35.55 ± 2.57Ba	34.16 ± 2.14Ba	33.28 ± 1.92Ba	1.86 ± 0.57ABCa	46.82 ± 1.00ABa	48.69 ± 1.42ABa	19.67 ± 0.93Ca		21.64 ± 2.79Aa
	Z	1.39 ± 0.06Aa	34.62 ± 2.78Ba	32.48 ± 2.62Ba	31.95 ± 2.68Ba	2.97 ± 0.38ABa	45.18 ± 1.89ABa	48.15 ± 1.91ABa	20.57 ± 1.67Ca		19.49 ± 2.84Aa
	SL	1.30 ± 0.07ABa	36.66 ± 1.72Ba	34.01 ± 1.28Ba	33.26 ± 1.24Ba	3.37 ± 1.02Aa	44.43 ± 2.44ABa	47.80 ± 1.71ABa	21.73 ± 0.99BCa		19.44 ± 1.63Aa
	ZL	1.24 ± 0.03Ba	42.07 ± 1.89Aa	39.76 ± 1.60Aa	38.20 ± 1.41Aa	2.83 ± 0.82ABa	49.34 ± 1.76Aa	52.18 ± 1.74Aa	21.48 ± 1.40BCa		25.53 ± 1.29Aa
	CK	1.34 ± 0.01ABa	33.35 ± 3.29Ba	32.24 ± 3.13Ba	31.62 ± 2.88Ba	1.48 ± 1.00BCa	43.23 ± 4.26ABa	44.72 ± 4.42Ba	26.70 ± 0.82Aa		8.90 ± 3.52Ba
	*P*	0.132	0.025	0.035	0.059	0.044	0.123	0.038	0.013		0.001
0–20	X	1.24 ± 0.09B	35.14 ± 4.30B	34.01 ± 3.86AB	33.22 ± 3.37AB	1.38 ± 0.62 C	42.13 ± 4.24 C	43.52 ± 4.69 C	26.95 ± 2.94 A		10.29 ± 5.00 C
	L	1.35 ± 0.08 A	35.34 ± 3.67B	33.63 ± 3.19AB	32.72 ± 2.91AB	2.24 ± 1.05ABC	45.31 ± 2.55ABC	47.56 ± 2.79ABC	20.09 ± 0.80 C		20.29 ± 4.26AB
	Z	1.33 ± 0.08 A	37.95 ± 4.09AB	35.28 ± 3.46AB	34.70 ± 3.46AB	3.49 ± 0.99 A	46.72 ± 2.18AB	50.21 ± 2.57AB	21.01 ± 1.48BC		22.29 ± 3.68AB
	SL	1.27 ± 0.07AB	36.69 ± 3.20AB	34.33 ± 2.40AB	33.41 ± 1.95AB	2.94 ± 1.31AB	43.48 ± 2.96BC	46.43 ± 3.57BC	22.84 ± 1.56B		17.54 ± 3.49B
	ZL	1.27 ± 0.05AB	40.24 ± 2.59 A	37.81 ± 2.37 A	36.30 ± 2.27 A	3.05 ± 0.92AB	47.91 ± 2.01 A	50.96 ± 1.98 A	21.94 ± 1.41BC		23.08 ± 2.91 A
	CK	1.34 ± 0.03 A	34.37 ± 2.82B	32.91 ± 2.48B	32.19 ± 2.25B	1.95 ± 1.31BC	44.35 ± 3.34ABC	46.31 ± 3.67BC	25.51 ± 1.35 A		11.90 ± 4.19 C
	*P*	0.034	0.098	0.159	0.211	0.023	0.030	0.007	0.000		0.000

Values are the mean ± SD. Significant differences (*P* < 0.05) among study sites are indicated by different uppercase letters, while significant differences (*P* < 0.05) between the upper layer and the lower soil layer within a study site are indicated by different lowercase letters. The *P* value is used to assess the statistical significance of differences, with *P* < 0.05 indicating significant differences.

In the upper soil layer, soil BD was lower in X and SL as compared to CK. The SHC, CHC, FHC, NCP, CP, and Pt did not differ between CK and the other study sites. Compared to CK, the soil MCg was lower in L, Z and ZL and higher in X; meanwhile, Ae was higher in Z than CK. Comparing among forested sites, soil BD was lower in X versus L and ZL, while Ae was lower in X versus L, Z and ZL; in contrast, the soil MCg was lowest in L and Z compared to the other forest types. In the lower soil layer, soil BD was lower in ZL as compared to CK, while SHC, CHC, FHC, Pt and Ae were higher; the soil MCg was lower in L, Z, SL and ZL than CK, while Ae was higher. Comparing among forested sites, ZL had a greater water holding capacity than the other forest types. Meanwhile, the soil Ae and NCP were lowest in X among all forested sites, while the soil MCg was highest.

When soil physical characteristics were averaged across both soil layers, the soil BD was lower in X as compared to CK. Meanwhile, the SHC, CHC, FHC, Pt and Ae were higher in ZL versus CK, and the soil MCg was lower in L, Z, SL and ZL versus CK, while Ae was higher. Comparing among forested sites, the soil BD, NCP, CP, Pt and Ae were lowest in X, while the soil MCg was highest in X; the SHC, CHC, FHC, CP, Pt and Ae were greatest in ZL.

Comparing soil physical characteristics between soil layers within study sites, significant differences were observed for soil BD in X, as well as for CHC, FHC, CP and Ae in ZL, where values were higher in the lower versus upper soil layer.

Soil chemical characteristics and two biological characteristics were shown in Table 3. All characteristics except for TK varied significantly among study sites, either within a specific soil layer or across both soil layers (i.e., in the averaged values). Overall, forest type affected the soil pH, appropriate nutrient and the microbial biomass in surface soils.

**Table 3:** Soil chemical and biological characteristics in forest shelterbelts of different types.

Layer/cm	Sample plot	pH	TOC/g๒kg^− 1^	AN/mg๒kg^− 1^	NN/mg๒kg^− 1^	TN/g๒kg^− 1^	AP/mg๒kg^− 1^	TP/g๒kg^− 1^	AK/mg๒kg^− 1^	TK/g๒kg^− 1^	MBC/mg๒kg^− 1^	MBN/mg๒kg^− 1^
0–10	X	6.75 ± 0.06Ca	25.57 ± 4.39ABCa	8.51 ± 3.64Aa	0.24 ± 0.03Da	2.19 ± 0.30ABa	28.79 ± 2.99Ba	0.43 ± 0.01Ca	524.22 ± 130.17Aa	12.70 ± 0.62Aa	162.83 ± 39.36Ba	107.24 ± 28.69Aa
	L	6.90 ± 0.08Ba	23.54 ± 1.84BCa	4.78 ± 0.41Ba	0.61 ± 0.16Ca	1.89 ± 0.18BCa	39.78 ± 0.81Aa	0.32 ± 0.01Da	270.06 ± 9.13BCa	12.21 ± 0.06Aa	107.66 ± 4.69Ca	37.61 ± 6.55BCa
	Z	6.71 ± 0.02CDa	20.71 ± 2.07Ca	2.41 ± 0.26Ba	1.59 ± 0.01Aa	1.67 ± 0.15Ca	20.43 ± 4.02Ca	0.31 ± 0.03Da	230.31 ± 29.40BCa	9.92 ± 1.47Aa	114.78 ± 11.64Ca	32.16 ± 6.99BCa
	SL	6.53 ± 0.12Ea	25.04 ± 0.92ABCa	4.00 ± 0.41Ba	0.83 ± 0.09Ca	2.05 ± 0.14BCa	19.60 ± 0.16Ca	0.34 ± 0.02Da	316.99 ± 17.72Ba	11.41 ± 2.81Aa	122.91 ± 13.06BCa	19.42 ± 4.47Ca
	ZL	6.58 ± 0.03DEb	27.64 ± 0.25ABa	3.19 ± 0.56Ba	0.70 ± 0.06Ca	2.29 ± 0.07ABa	21.92 ± 1.58Ca	0.50 ± 0.01Ba	180.83 ± 41.06CDa	11.65 ± 0.14Aa	144.93 ± 17.21BCa	38.93 ± 7.47BCa
	CK	8.30 ± 0.03Aa	29.25 ± 1.86Aa	2.09 ± 0.09Ba	1.19 ± 0.15Ba	2.52 ± 0.14Aa	37.63 ± 1.91Aa	0.57 ± 0.03Aa	66.98 ± 4.90Da	11.10 ± 0.89Aa	218.01 ± 10.55Aa	59.22 ± 4.67Ba
	*P*	0.000	0.036	0.013	0.000	0.007	0.000	0.000	0.000	0.467	0.001	0.000
10–20	X	6.61 ± 0.02Cb	20.00 ± 1.31Ba	5.10 ± 1.08Aa	0.10 ± 0.02Db	1.77 ± 0.12Ba	29.71 ± 4.88ABa	0.35 ± 0.03Bb	249.38 ± 3.37Ab	10.19 ± 1.40Ba	114.24 ± 11.51ABa	68.99 ± 16.67Aa
	L	6.87 ± 0.11Ba	21.79 ± 3.85Ba	4.48 ± 0.99ABa	0.40 ± 0.03Ca	1.73 ± 0.29Ba	34.49 ± 1.63Ab	0.33 ± 0.06Ba	244.48 ± 25.16Aa	12.01 ± 0.20ABa	91.32 ± 8.89Ba	13.52 ± 3.78Cb
	Z	6.83 ± 0.12BCa	20.87 ± 1.46Ba	3.03 ± 1.33BCa	1.56 ± 0.03Aa	1.71 ± 0.14Ba	20.27 ± 0.65Ba	0.37 ± 0.03Ba	202.43 ± 15.01Ba	11.46 ± 0.78ABa	108.63 ± 20.77ABa	38.06 ± 10.81Ba
	SL	6.77 ± 0.10BCa	21.99 ± 0.34Bb	2.69 ± 0.29BCb	0.39 ± 0.06Cb	1.80 ± 0.01Ba	20.61 ± 1.37Ba	0.37 ± 0.01Ba	209.40 ± 12.55Bb	12.67 ± 0.81Aa	107.90 ± 14.77ABa	15.00 ± 3.66Ca
	ZL	6.81 ± 0.11BCa	26.49 ± 0.68Aa	2.32 ± 0.14Ca	0.33 ± 0.05Cb	2.17 ± 0.05Aa	26.50 ± 5.40ABa	0.47 ± 0.00Aa	56.49 ± 0.83Cb	11.88 ± 0.24ABa	122.22 ± 8.32ABa	41.06 ± 1.17Ba
	CK	8.46 ± 0.09Aa	26.82 ± 0.70Aa	2.29 ± 0.13Ca	0.63 ± 0.03Bb	2.21 ± 0.02Ab	23.31 ± 7.59Ba	0.48 ± 0.02Ab	55.65 ± 3.90Ca	11.36 ± 1.02ABa	138.26 ± 23.72Ab	58.16 ± 6.98ABa
	*P*	0.000	0.009	0.019	0.000	0.010	0.045	0.002	0.000	0.170	0.153	0.000
0–20	X	6.68 ± 0.08 C	22.79 ± 4.27B	6.81 ± 3.18 A	0.17 ± 0.07D	1.98 ± 0.31BC	29.25 ± 4.07B	0.39 ± 0.04B	386.80 ± 165.41 A	11.45 ± 1.66 A	138.54 ± 37.83B	88.12 ± 30.27 A
	L	6.88 ± 0.10B	22.67 ± 3.14B	4.63 ± 0.77B	0.51 ± 0.16 C	1.81 ± 0.25CD	37.14 ± 2.94 A	0.32 ± 0.04 C	257.27 ± 22.84B	12.11 ± 0.18 A	99.49 ± 10.83 C	25.56 ± 13.18CD
	Z	6.77 ± 0.10BC	20.79 ± 1.79B	2.72 ± 1.01BC	1.57 ± 0.03 A	1.69 ± 0.15D	20.35 ± 2.88 C	0.34 ± 0.04BC	216.37 ± 27.19BC	10.69 ± 1.40 A	111.71 ± 17.11BC	35.11 ± 9.56CD
	SL	6.65 ± 0.16 C	23.51 ± 1.68B	3.35 ± 0.75BC	0.61 ± 0.23 C	1.93 ± 0.16CD	20.11 ± 1.10 C	0.36 ± 0.02BC	263.20 ± 55.94B	12.04 ± 2.16 A	115.41 ± 15.83BC	17.21 ± 4.64D
	ZL	6.69 ± 0.14 C	27.07 ± 0.77 A	2.76 ± 0.60BC	0.52 ± 0.19 C	2.23 ± 0.08AB	24.21 ± 4.59BC	0.48 ± 0.02 A	118.66 ± 68.62CD	11.77 ± 0.23 A	133.54 ± 17.65BC	40.00 ± 5.45BC
	CK	8.38 ± 0.10 A	28.04 ± 1.86 A	2.19 ± 0.15 C	0.91 ± 0.30B	2.36 ± 0.19 A	30.47 ± 9.05B	0.53 ± 0.05 A	61.32 ± 7.19D	11.23 ± 0.97 A	178.14 ± 43.90 A	58.69 ± 5.96B
	*P*	0.000	0.000	0.000	0.000	0.000	0.000	0.000	0.000	0.533	0.001	0.000

Values are the mean ± SD. Significant differences (*P* < 0.05) among study sites are indicated by different uppercase letters, while significant differences (*P* < 0.05) between the upper layer and the lower soil layer within a study site are indicated by different lowercase letters. The *P* value is used to assess the statistical significance of differences, with *P* < 0.05 indicating significant differences.

In the upper soil layer, the pH, TOC, TN, TP and MBC were lower in the forested sites versus CK, while AN and AK were higher; however, only the differences in pH, TP and MBC were significant. Comparing among forested sites, soil NN was lowest in X, while AN, AK and MBN were highest. Meanwhile, the pH and AP were lower in Z, SL and ZL compared to other sites, and TP was lower in Z and SL. Within the monoculture shelterbelts, the soil AN, AK and MBN were higher in X versus L or Z, while NN was lower, and relatively slight decreased MBC.

In the lower soil layer, the pH, TOC, TN, TP and MBC were generally lower in the forested sites versus CK, while AN was higher; however, only the differences in pH was significantly lower than CK. Comparing among forested sites, the soil pH, TOC, NN and TK were lowest in X, while MBN was highest. Meanwhile, soil AP was highest, while MBC and MBN were lowest in L; soil NN was highest in Z. Soil NN, AP, AK and MBN significantly differed between L and Z. Between the two mixed forests, SL and ZL, soil TOC, TN, TP, AK and MBN differed significantly.

When the soil characteristics were averaged across both soil layers, the soil pH, TOC, TN, TP and MBC were lower in the forested sites versus CK; however, these differences were only significant for pH and MBC. Meanwhile, soil AN and AK were visibly higher in the forested sites versus CK, especially in X, L and SL. Comparing among forested sites, soil AN, AK, MBC and MBN were highest in X, while soil NN was lowest. Soil TP and MBC trended lower in L than the other forested sites. Soil NN was significantly higher in Z versus the other sites, but TN was generally lower. The soil pH and MBN were lowest in SL, while TOC, TN and TP were highest in ZL. Comparing soil chemical and biological characteristics between soil layers within study sites, the pH, NN, TP, AK of X and AP in L; TOC, AN, NN and AK in SL; NN and AK in ZL; and AN, TN and TP in CK were all significantly lower in the lower soil layer than in the upper soil layer. Meanwhile, the pH in ZL showed the opposite pattern. In the forested sites, soil NN, AK, and MBC were significantly higher in the upper soil layer compared to the lower soil layer.

### Characteristics of soil bacterial communities across study sites

For all 36 samples (six per study site), amplified PCR products were sequenced using a paired-end protocol and sequencing reads then quality filtered; a total of 1,418,914 sequences were obtained, an average of 39,414 per sample, of which 99.93% were between 400 bp and 450 bp in length. Sequences were assigned to OTUs at 97% sequence similarity, and a total of 121,164 OTUs were obtained. After removing rare OTUs, 10,687 OTUs remained, and after controlling for sequencing depth, the number of OTUs was further reduced to 10,184.

**Fig. 2:**
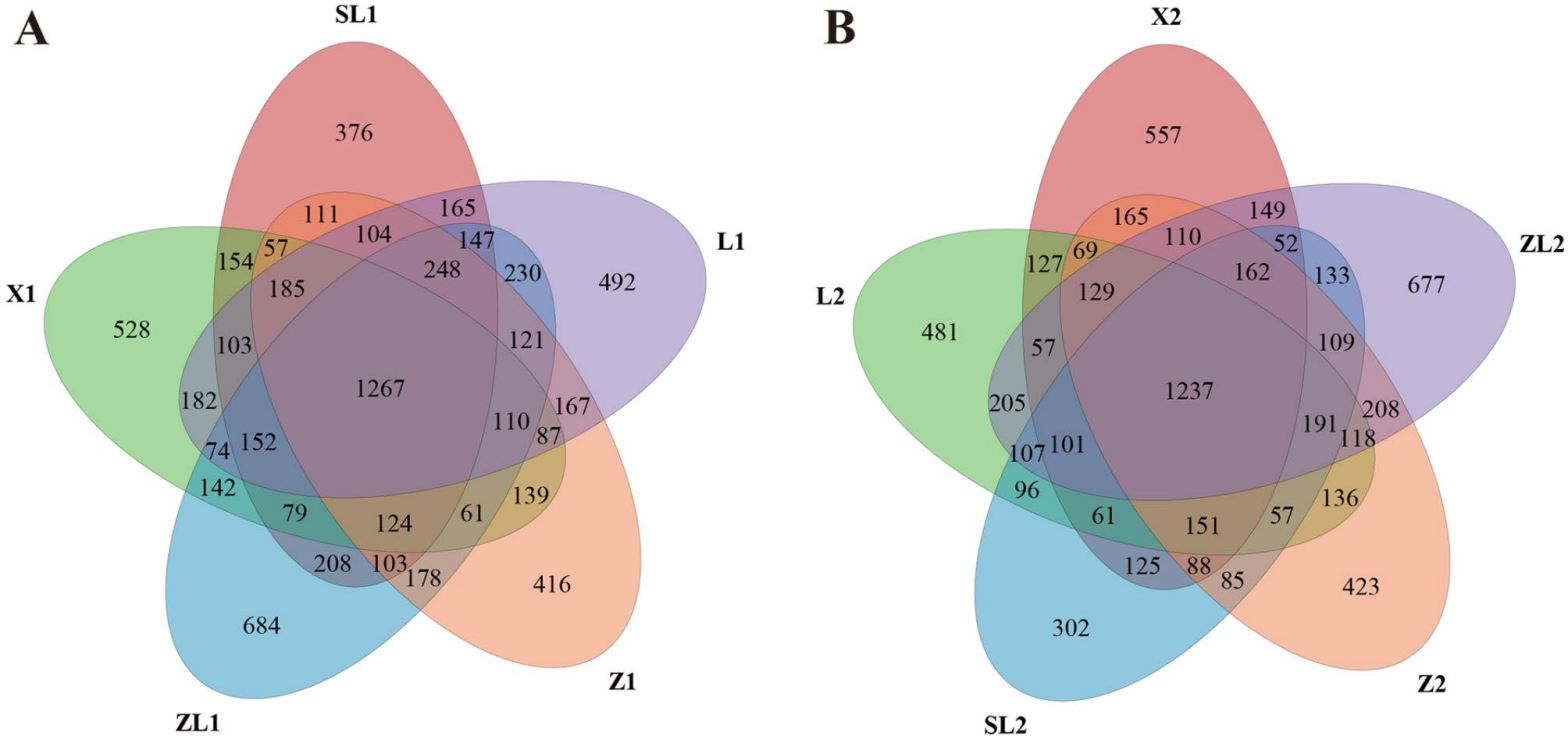
Venn diagrams of OTUs shared and unique among forest types.

Based on the OTU occurrence data, Venn diagrams were drawn to assess the number of shared OTUs and unique OTUs in the upper and lower soil layers (Fig. 2A and B, respectively) for each study site. In the upper soil layer, a total of 1267 OTUs were found in all forested sites, or 17.61% of the total number of OTUs observed across all forested sites. For X1, L1, Z1, SL1 and ZL1, unique OTUs accounted for 7.34%, 6.84%, 5.78%, 5.23% and 9.51% of the total, respectively. Soils in ZL1 supported the most unique soil bacterial diversity, while soils in SL1 supported the least. Among the forested sites, ZL1 contained the most OTUs (3928) overall and X1 the least (3444). Comparing the number of unique OTUs to the total number observed in each forested site, the proportion of unique OTUs was highest in ZL1 (17.41%) and lowest in SL1 (10.49%). In the lower soil layer, a total of 1237 OTUs were found in all forested sites, or 18.55% of the total number of OTUs observed across all forested sites. For X2, L2, Z2, SL2 and ZL2, unique OTUs accounted for 8.35%, 7.21%, 6.34%, 4.53% and 10.15% of the total, respectively. Soils in ZL2 also supported the most unique soil bacterial diversity, while soils in SL2 supported the least. Comparing forested sites, ZL2 contained the most OTUs overall (3745) and SL2 the fewest (3057). The proportion of total OTUs accounted for by unique OTUs was greatest in ZL2 (18.08%) and smallest in SL2 (9.88%).

Across all study sites, the number of OTUs was higher in the upper soil layer versus the lower soil layer. Site SL1 showed the greatest difference in the number of OTUs between soil layers (14.68% fewer OTUs in the lower layer), while Z showed the smallest difference (1.15%). Soil bacteria were therefore more abundant in the upper soil layer for all forested sites, and this difference was significant. However, the magnitude of this difference in OTU richness varied among sites: examining unique OTUs, X and Z had 29 and 7 more unique OTUs in the lower soil layer, respectively, while L, SL and ZL had 11, 74 and 7 fewer unique OTUs, respectively.

### Comparison of soil bacterial communities among forest types

**Fig. 3:**
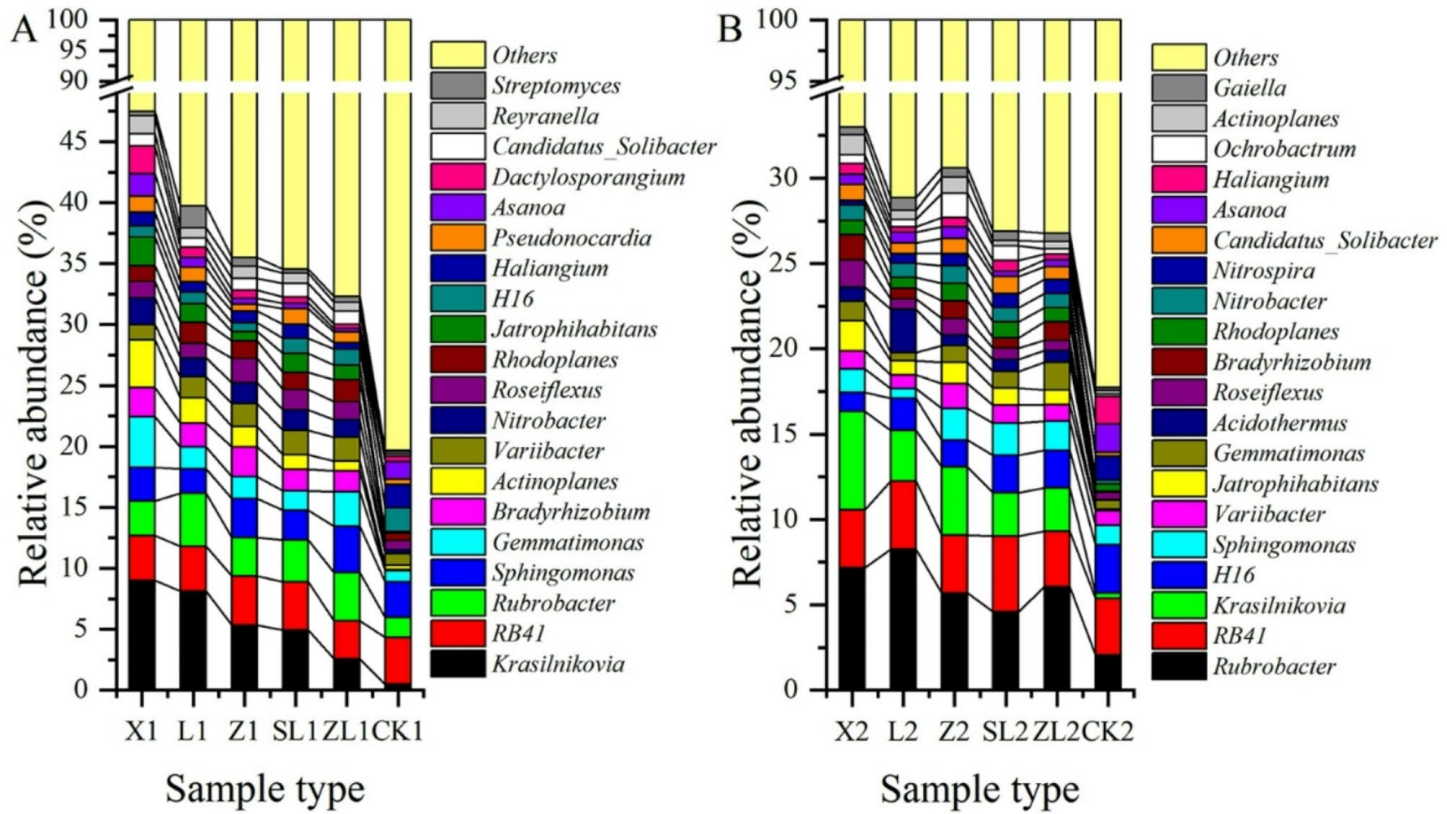
Abundance histogram for the 20 most abundant soil bacterial genera.

Representative sequences from each study site were compared with the Silva database to obtain the abundance distribution for the 20 most common genera (Fig. 3). In the upper soil layer, the number of genera (with abundance > 1%) was higher in the forested sites (X, *n* = 18; SL, 16; L, 14; ZL, 13; and Z, 11) than CK (*n* = 6); *Krasilnikovia*, *RB41*, *Rubrobacter* and *Sphingomonas* were the most common genera in forest soils (abundance > 2.00%). In addition to these genera, *Gemmatimonas*, *Actinoplanes*, *Bradyrhizobium*, *Nitrobacter*, *Jatrophihabitans* and *Dactylosporangium* were most common in X; *Actinoplanes* in L; *Bradyrhizobium* in Z; *Variibacter* in SL; *Gemmatimonas* in ZL; and *RB41* and *Sphingomonas* in CK. In the lower soil layer, the number of genera (abundance > 1%) was the same in X and Z (*n* = 11), the same in SL, ZL and CK (7), and lowest in L (6); *Rubrobacter*, *RB41* and *Krasilnikovia* were the most common genera in forest soils (abundance > 2.00%). In addition to these genera, *Acidothermus* was the most common genus in L; *H16* in SL and ZL; and *Rubrobacter*, *RB41* and *H16* in CK. Comparing the upper and lower soil layers, there were more dominant genera in the upper soil layer, and the relative abundance of the same dominant genera also differed.

### Relationships between soil physicochemical characteristics and soil bacterial genera

**Fig. 4:**
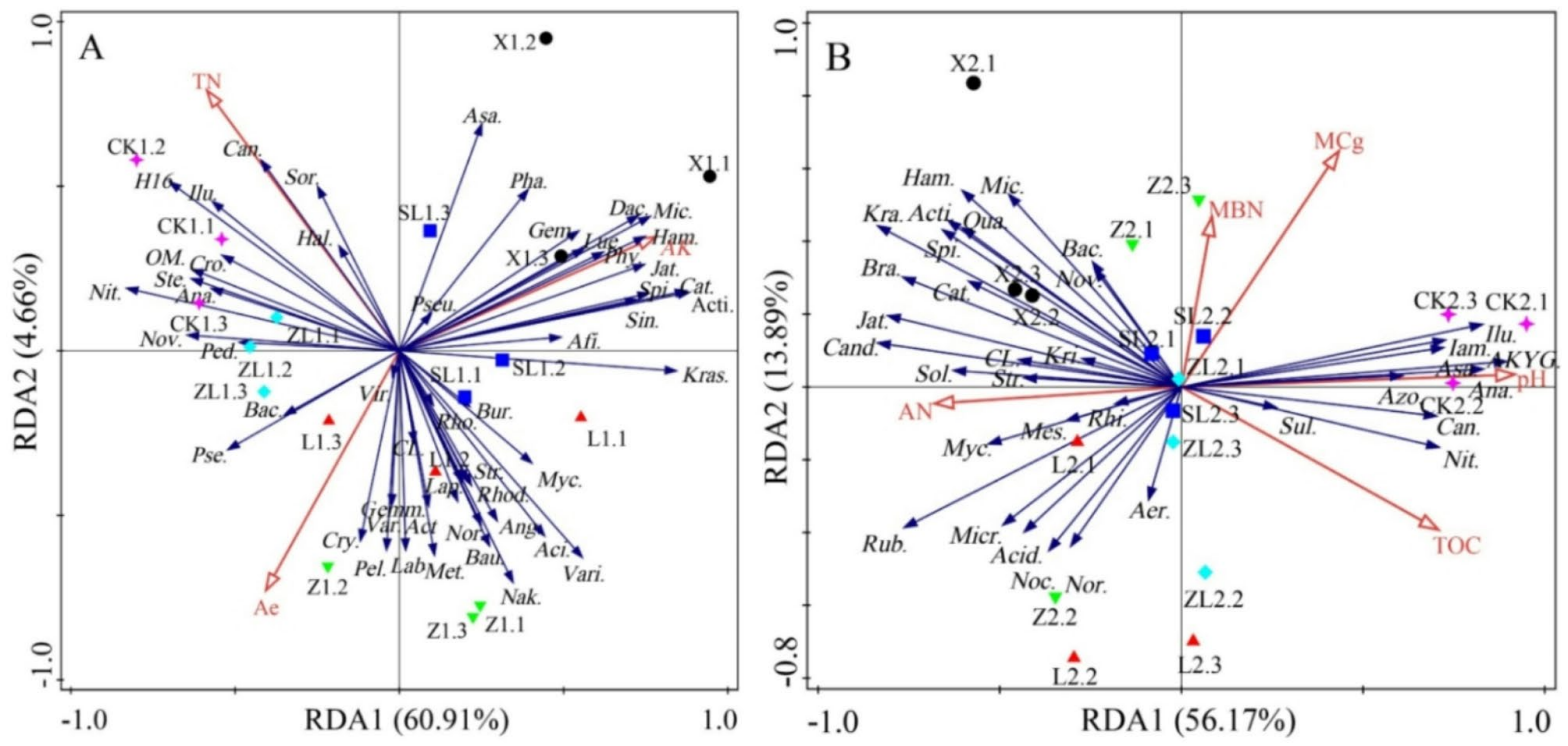
RDA based on soil physicochemical characteristics and bacterial genera.

An RDA was performed based on abundance data for the soil bacterial genera that differed in abundance among study sites and the soil physicochemical characteristics, and an ordination diagram of the first two axes was produced (Fig. 4). For the upper soil layer analysis, the 14 soil physicochemical characteristics were included in the RDA model and forward selection was used. Using the forward selected RDA conditional effects, characteristics showing collinearity were identified and removed (variance inflation factor, *VIF* > 10), and the following characteristics (contribution > 10% or *P* < 0.05) used to repeat the ordination (Fig. 4A): AK (41.2%, *P* = 0.006), TN (21.3%, *P* = 0.004) and Ae (10.5%, *P* = 0.026). In this new RDA model (adjusted *R*^*2*^ = 60.3%), all canonical axes were significant (*Trace* = 0.673, *F* = 9.602, *P* = 0.002); together, the first (60.91%) and second (4.66%) axis explained 65.57% of the variance. The soil characteristics AK, TN and Ae accounted for 38.0% (*Pseudo-F* = 9.8, *P* = 0.004, *VIF* = 1.115), 19.6% (*Pseudo-F* = 6.9, *P* = 0.004, *VIF* = 1.091) and 9.7% (*Pseudo-F* = 4.2, *P* = 0.028, *VIF* = 1.173) of the variance, respectively.

For the lower soil layer analysis, the eleven soil physicochemical characteristics were included in the RDA model and forward selection was used. Using the forward selected RDA conditional effects, collinear characteristics were removed (variance inflation factor, *VIF* > 10), and the RDA repeated (Fig. 4B) with the following soil physicochemical characteristics (contribution > 10% or *P* < 0.05): pH (57.6%, *P* = 0.002), MCg (10.4%, *P* = 0.032), AN (11.0%, *P* = 0.014) and TOC (7.0%, *P* = 0.034). In the new RDA model (adjusted *R*^*2*^ = 64.2%), all canonical axes were significant (Trace = 0.762, *F* = 7.678, *P* = 0.002); together, the first (56.17%) and second (13.89%) axis explained 70.06% of the variance. Individually, the soil pH, AN, MCg and TOC accounted for 48.6% (Pseudo-*F* = 15.1, *P* = 0.002, *VIF* = 2.169), 9.3% (Pseudo-*F* = 3.9, *P* = 0.008, *VIF* = 1.620), 8.8% (Pseudo-*F* = 3.1, *P* = 0.032, *VIF* = 1.533) and 5.9% (Pseudo-*F* = 2.8, *P* = 0.050, *VIF* = 1.959) of the variance, respectively.

## Discussion

Afforestation significantly impacts ecosystem biogeochemical processes^36^, altering soil characteristics^37^ and affecting the structure and function of soil microbial communities^38^.

### Effects of shelterbelt forest type on soil physicochemical characteristics

In this study, forest type significantly affected most surface soil characteristics, except for SHC, CHC, FHC, and TK, following previous work^9^. Compared to CK, shelterbelt soils had greater SHC, CHC, and FHC, meaning that afforestation improved soil water holding capacity, consistent with Fang and Wu^39^. Shelterbelts were characterized by a thicker litter layer, which likely contributed to loosening the topsoil, this capacity loosened the topsoil, reducing BD, as seen by Wu^10^. Among study sites, the soil BD was lowest in X, which also had the most developed forest root system. Interestingly, though transpiration rates are known to be very high in X^40^, the soil MCg was higher in X than in CK; This apparent contradiction may reflect root-mediated water uptake from deeper soil layers combined with reduced evaporation caused by lower soil Ae in X. Specifically, X exhibited the lowest Ae but the highest Pt and MCg across all forest types. For X, the soil BD was reduced in the upper soil layer versus the lower soil layer, which may be related to the high MCg observed in the upper soil layer. To some extent, the greater the MCg, the smaller the BD will be. Examining ZL, soil CHC, FHC, CP, and Ae were consistently higher in the lower soil layer than in the upper layer. Compared to other forest types (X, L, Z, SL), ZL also exhibited superior water-holding capacity and aeration in both individual soil layers and averaged across depths. Lower soil MCg in L, Z, SL, and ZL were likely related to the relatively short root system and high permeability of coniferous tree species, while higher soil Ae could be explained by the abundance of capillary roots and surface litter. Surface soils had the greatest water holding capacity in ZL, potentially related to the thick litter layer at this site and high soil Pt and Ae. TK in black soils primarily originates from primary minerals (e.g., orthoclase, plagioclase; 90–98% contribution). As mineral weathering is slow, 20-year afforestation had no significant impact on TK levels^41^. However, shelterbelts reduced surface soil pH compared to CK, likely driven by decreased groundwater levels (from tree transpiration) and leaching of soluble alkali metals^42^. Given that the study shelterbelts were all planted on similar substrates, differences in the tree species planted and resultant forest litter have produced the observed differences in soil pH. This is consistent with Iovieno^43^, who found that varying the dominant species in stands on the same substrate differentially affected soil pH. Here, afforestation led to decreases in TOC, TN, TP and MBC, with MBC showing the most substantial decline, different from previous studies^33,37^. This discrepancy may stem from the fact that the study shelterbelts were narrow bands that often suffered from wind erosion, which was not conducive to the accumulation of leaf litter and reduced the abundance of understory herbaceous plants. The establishment of the shelterbelts altered soil materials and energy flowing, as well as the soil development process, and led to the difficulty in maintaining the balance of soil nutrient cycles. The effects of afforestation on soil C, N, P, K, MBC and MBN were inconsistent across study sites, a pattern previously observed by other authors^33,37^. Compared to CK, forested sites had higher AN, likely as the result of organic matter mineralization in the soil, which corresponded with decreases in TOC. Comparing soil layers, NN was generally reduced in the lower soil layer versus the upper soil layer, likely related to differences between soil layers in AN and nitrification conditions (the abundance of nitrifying bacteria, etc.). Higher levels of NN in surface soils could be driven by plant roots absorbing water from deeper soil layers, as NN is soluble and would migrate with the water. Similar tree-driven patterns in available nutrients have also been found in previous studies^8,44^. The accumulation of AK in surface soils was most likely caused by the large amounts of plant ash deposited on the soil surface as a result of the incineration of crop straw by local farmers. Meanwhile, the surface accumulation of MBC was probably driven by higher microbial biomass in the upper soil layer.

### Soil microbial responses to shelterbelt tree species composition

In this study, the total number of soil bacterial OTUs differed between the upper and lower soil layer, and was higher in the forested sites as compared to CK, suggesting that farmland shelterbelts increased soil bacterial diversity. The number of OTUs was highest in ZL, perhaps because its litter layer was thicker than that of the other forested sites (litter was absent in X). Mixed species shelterbelts tend to have more heterogeneous soil microbial communities than monocultures^9^, likely because more complex habitats lead to diverse microbial communities^45^, as microorganisms have differing physiological and evolutionary adaptations to the environment^46^. While SL1 did not have the smallest number of OTUs, it did have the lowest proportion of unique OTUs, indicating that SL1 shared many OTUs with the other forested sites. Both the abundance of dominant genera and the total number of OTUs were higher in the upper versus lower soil layer, a phenomenon also found by Carnovale, Bissett, Thrall and Baker^9^. Afforestation also shifted bacterial community composition: forest sites hosted higher relative abundances of dominant genera (e.g., *Krasilnikovia* from Actinobacteria) but lower evenness compared to CK. Similar depth- and vegetation-dependent patterns were observed in European forests^47,48^, though dominant taxa varied regionally—likely due to differences in soil pH and TOC. For instance, acidic soils (pH 4.2–4.9) in the Greater Hinggan Mountains favored Acidobacteria and Proteobacteria^49^, whereas neutral-to-alkaline black soils (pH 6.53–8.46) in this study promoted Actinobacteria dominance. Declines in TOC following afforestation here may further explain these taxonomic divergences^50^.

Notably, X exerted the strongest influence on bacterial communities, hosting the most genera with differential abundance across sites. As the forest in X had the most extensive root system, its activity may help explain this effect; more root activity increases soil organic matter, altering the soil habitat for bacterial communities^46^. This finding is similar to that of Jiang^51^, who found greater soil microbial diversity in broad-leaved and mixed broad-leaved forests than in coniferous forests. The prevalence of Actinobacteria in X may also reflect decomposition of aging roots, while Proteobacteria dominance in CK correlated with higher C, N, and P levels, underscoring their role as nutrient status indicators^44^. The abundance of Actinomycetes and Proteobacteria varied among study sites, consistent with previous studies^52^. Lauber^38^ determined that Acidobacteria were most prevalent in acidic soils. In this study, more genera varied in abundance with the study site in the upper soil layer than in the lower soil layer, consistent with the fact that the upper layer also contained more OTUs.

### Factors influencing soil bacterial communities across study sites

In this study, the factors influencing community composition differed between soil layers. In the upper soil layer, AK was the most influential factor, followed by TN and Ae. Recently, sites have received significant surface deposits of ash from straw burning, which may explain the adaptation of soil bacterial communities to high AK environments. Surface soils experience variation in organic nitrogen levels, as rates of nitrogen leaching and respiration vary among forests. Resultant differences in TN and Ae provide heterogeneous environments for the growth of soil bacterial communities^53^. In contrast to these results, Deng^22^ identified different soil characteristics as the most influential factors affecting soil bacterial communities. They studied large forest plantations and found that soil C and N were the primary drivers of bacterial community composition, likely due to differences in forest type and overall soil characteristics. In the lower soil layer, soil pH was the most influential factor, with AN, MCg, and TOC also contributing significantly. Soil pH correlated with the abundance of 15 bacterial genera, including Acidothermus, which showed a negative relationship—consistent with its acidophilic nature^22,54^. While pH strongly governs bacterial diversity in Northeast China’s black soils, ancillary effects of C, N, and MCg were also evident^21^. Inorganic matter-rich black soils, carbon, and nitrogen are often positively correlated, but nitrogen is the limiting factor for soil microorganisms^44^. In contrast to Yang^33^, who identified different soil characteristics underlying patterns of bacterial diversity; however, they used stoichiometric ratios among soil characteristics for their RDA analysis, while the absolute values of soil characteristics were used in this study. Besides, soils were acidic in Yang, but neutral or weakly alkaline here. Previously, TOC has been found to affect the soil MCg^55^, but this relationship was not found here (Fig. 3B); in the lower soil layer, MCg and TOC had different effects on the bacterial community.

On the whole, tree species identity not only affected soil physicochemical characteristics in the afforested sites, but also the soil bacterial community. At the same time, soil bacterial communities were affected by the soil physicochemical characteristics. Variation across study sites in the soil bacterial community was a comprehensive response to both soil physicochemical characteristics and tree species effects.

### Implementations

The findings suggest that implementing mixed forests, particularly those comprising *Pinus sylvestris* and *Larix gmelinii*, can significantly enhance soil health and microbial diversity in shelterbelts. This information is valuable for land managers and policymakers aiming to optimize shelterbelt design for improved ecological benefits.

### Limitations

The study was conducted in a specific region (mid-west of Heilongjiang Province), and the results may not be fully generalizable to other regions with different climatic and soil conditions.

### Future perspectives

Future research should aim to include a broader range of shelterbelt types and regions to validate and extend these findings. Investigating soil microbial communities will provide a more comprehensive understanding of shelterbelt ecology.

## Conclusions

This study emphasizes the impact of different shelterbelt compositions on soil physicochemical properties, microbial biomass, and bacterial community diversity. The findings reveal that the *Pinus sylvestris* and *Larix gmelinii* mixed forest significantly enhances soil aeration, water holding capacity, and nutrient availability, thereby promoting higher microbial activity and diversity. These improvements in soil health and microbial diversity are crucial for the sustainability of farmland shelterbelts in the black soil region of China. The study highlights the pivotal role of available potassium and pH in shaping soil bacterial communities, emphasizing the need for targeted soil management practices that enhance these factors.

Overall, the research contributes to a better understanding of the interactions between tree species composition and soil microbial communities, offering practical recommendations for enhancing the ecological functions of shelterbelts. Future studies should build on these findings by exploring a broader range of shelterbelt types and regions, as well as incorporating long-term assessments to ensure the sustainability of these ecological benefits.

## Data Availability

Raw sequence data were deposited in the NCBI Sequence Read Archive database (SRA) with the access number SRP187158.
